# Observations on Puerperal Fever

**Published:** 1864-10

**Authors:** Delaskie Miller

**Affiliations:** Chicago


### CHICAGO MEDICAL EXAMINER.

N. S. DAVIS, M.D., Editor.

VOL. V. OCTOBER, 1864. NO. 10.

### Original Contributions.

### ARTICLE XXXIV.

### OBSERVATIONS ON PUERPERAL FEVER.

By DELASKIE MILLER, M.D., of Chicago.

Being a portion of the Report of the Committee on Obstetrics, read to the Illinois State Medical Society. May, 1864.

The prevalence of *Puerperal Fever* in certain localities within the State during the past year—the diversity of opinions entertained by the profession upon the nature of the disease—its communicability and its treatment, are reasons sufficient for making at least a brief allusion to it, in the report of your Standing Committee on Obstetrics.

More than ordinary interest attaches to the history of this disease, from the fatality which has attended its prevalence wherever it has appeared in an epidemic form, and the antagonistic views which have been entertained in regard it. And yet these reasons would not justify your Committee in occupying the time of this meeting, with considerations relating to it, if the leading minds in the profession were, at the present day, agreed or nearly in harmony in regard to two important points, viz.: its communicability from the attendant or others, to the parturient, and upon the most successful plan of treatment. But, unfortunately, upon these points the most opposite opinions are still held and taught by medical men.

The terms employed to characterize the disease, as it has prevailed at different times and places, give us some idea of its severity, such as "fatal malady"—"terrible"—its name is "a word of fear"—"appalling," &c., and the statistics which have descended to us, prove that these phrases were not the expressions of a poetical fancy. "While mortality in London is about 1 in 150, and in Lying-in Hospitals varies from 1 in 70 to 1 in 100, the mortality in the Hotel Dieu and in the Maternite was 1 in 20, sometimes 1 in 13, chiefly caused by puerperal fever. The mortality in the great Vienna Hospital was as high as 1 in 10, and even 1 in 6."

In London, in 1761, the mortality was so great that in some of the smaller Lying-in Hospitals, they buried two women in one coffin to conceal their loss. The course of the disease was similar in most other cities of Great Britain and the Continent; the statistics of which would be out of place here, by taking too much space, without elucidating any practical principles.

A more interesting, and possibly useful, object would be attained by passing in hasty review, the diverse opinions which have at different times been entertained of its nature, and the various modes of treatment which have been adopted therefrom.

Strother, in 1716, was the first to apply the epithet "*Puerperal Fever*" to this disease, but there is reason to doubt whether he entertained more correct or definite ideas of the nature of the disease, than had his predecessors, who under the general term "*Child-bed Fever*," were in the habit of referring to most of the diseases of the puerperal state.

The disease broke out as an epidemic in Paris, in 1746; and at the Hotel Dieu it prevailed with such fatality, that it is asserted scarcely any recovered, and from this time we may date our first reliable history of it. Malouin has given an accurate and faithful account of this epidemic. Tenon's description, given many years later, states that the disease had become naturalized, for from 1774 to 1816, seven out of every twelve women delivered were seized with the disease.

Among the earliest accounts of the treatment adopted in the disease, we find that of Doulcet, who relied entirely on emetics, and it is affirmed saved several patients, and upon this he supposed that he had discovered the specific. This is not the only instance in the history of medicine in which subsequent experience has failed to sustain the claims of discoverers.

In 1768, DENMAN advised depletion on the first onset of the attack, and tartar emetic to cause vomiting afterwards.

Dr. HULME, in 1772, claimed the honor of discovering the cause of puerperal fever. He considered inflammation and gangrene of the omentum, as the essential disease, with some deterioration of the blood. He observes, "But the most capital of all remains, I mean, to cut off the purulent fomes, the chief cause of the disease, and to restore the tainted omentum and intestines to somewhat of their perfect state."

Dr. GORDON, of Aberdeen, in 1792, believed the disease to be an inflammation, but of the erysipelatous type. He advoe cated strongly bold and early depletion, 20 or 24 oz. at once, and if necessary 10 more soon after. "When I took away," he says, "only 10 or 12 ℥ of blood from my patient, *she always died*—but when bled freely she never failed to recover."

Mr. HEY, in 1812, resorted to active purgatives, but these proving unavailing, he was induced to resort to Dr. GORDON'S plan, taking frequently from 30 to 40 oz. and even 50 oz. of blood, and it is affirmed with good success.

Dr. ARMSTRONG, in 1819, also followed the depleting plan, by venesection and calomel purges.

Dr. GOOCH relied on venesection, leeching, and cathartics.

In 1823, Dr. COPELAND was in charge of Queen Charlotte's Hospital during the prevalence of puerperal fever, and from the failure of the systems of treatment then in vogue, he adopted a *stimulating* plan. On the first appearance of the symptoms of the malady, he administered from 8 to 16 grs. of camphor, combined with from 10 to 20 grs. of calomel, and from 1 to 3 grs. of opium, and repeated this every 4, 5, or 6 hours. Soon after the second dose of the medicine had been taken, half an ounce of spirits of turpentine, with castor oil, was administered, the stimulent and anodyne being still continued, and counter irritants applied to the abdomen. The success of this course, he assures us, in the malignant form, was almost complete.

Without intending to elaborate the subject in all its phases, three topics will claim attention at the present time. The nature—the communicability—the treatment.

What is the nature of *Puerperal Fever?* Here we meet a most important question, for not only the treatment of the disease, but in numerous instances, the immunity of the patient from the disease, will depend upon the views which the physician may entertain. Is the disease essentially a phlegmasia? Changes are frequently met with in *post mortem* examinations, in some one part of the system or another, having the semblance of the results of inflammation, but they are not uniform in their location or appearance, and they are sometimes absent altogether, no one of which being constant. If the disease depends upon inflammation in some organ, of course, when the local inflammation is *absent*, the puerperal fever cannot be *present*.

The testimony of those who have had the largest experience in treating this disease, and the best opportunities to observe its effects upon the system concur, in one particular, viz.: that cases are met with in which no morbid appearances can be found in the tissues after death. Then common inflammation is not essential to the existence of the disease.

The disease may run its course with such rapidity as to destroy the patient in a few hours, like ARMSTRONG'S "congestive disease," and MACKINTOSH'S "latent peritonitis," but do not terms like these deceive the observer? by leading him to suppose that he comprehends the case, while he remains innocent of the slightest idea of the true nature of the malady; are we not sustained in this supposition by what has been already collated? What could be more opposite than the depletion of GORDON, HEY, and ARMSTRONG, and the stimulation of COPELAND, each claiming to treat the same disease.

The fallacy consists in not recognizing a new element in puerperal fever, not found in ordinary inflammation, which renders its nature essentially different from peritonitis, phlebitis, metritis, &c., &c. The new element, we are led to believe, is a poison in the blood, producing its septic influence there—and through this medium producing changes sometimes in the tissues of important organs. That the disease is truly zymotic. Its history observes the laws of all poisons.

1st. It is an uniform disease; the description given of it an hundred years ago, describes the disease of to-day equally well.

2d. It selects a tissue for its seat, viz.:—the serous membranes and tissues analagous to them.

3d. The definite action is in the blood, the quantity of fibrine is increased, its quality is deteriorated.

4th. The action of the poison is modified by the quantity introduced into the circulation. When it is in excess the patient may die suddenly, without leaving any local manifestations of its presence. I am familiar with Dr. A. CLARK'S observations in regard to the valves at the extremities of the uterine sinuses, and the appearances of inflammation there in puerperal fever. But these appearances have not always been detected by other observers. When the poison is in less quantity its course is less rapid, and is followed by local changes.

By this view we are enabled to account for the diversity of opinions which have been promulgated of the nature of the disease. When partial or erroneous views are entertained of a disease, the observer will note the appearance in the individual case as representative of the class, and the account will therefore vary with each case examined, whereas, if the disease depends upon a *matires morbi* in the blood, accidental causes may determine in which organ, if any, the local changes are to be found.

It would be interesting to trace this poison to its source, and describe in detail the mode of its communication, but we can do little more than state conclusions.

1st. It may originate within the system, from the decomposition of organic matter.

2d. It may be introduced from without, by exposure to diseases characterized by ichoræmia, or,

3d. It may be communicated by the attendant, who is the vehicle of transportation from a distant case.

Notwithstanding evidence as irrefragible as can be produced, to prove the contagiousness of any disease, is abundant upon this, still it has been maintained in a most positive manner that *puerperal fever* is not communicated thus, and this position is defended by rare eloquence, and a personal experience of many years is referred to, as confirmatory of the position.

Suppose a physician in extensive practice is frequently called in consultation in cases of puerperal fever, and has not communicated the disease to his patients, what does it prove? Merely that a contagious disease is not necessarily taken by exposure. This is not peculiar to puerperal fever, for then would the prevalence of scarlatina, measles, small-pox, &c., rapidly become general upon the breaking out of the disease. No system could resist the power of the contagion; but what physician, that has practiced for any considerable time, has not known some of these diseases to affect a single member of a family, while all the others escaped. A single case coming within the knowledge of the reporter will be detailed briefly, as representative of many similar ones that might be collated from other sources.

A physician had been engaged to attend a lady in her first confinement, whose gestation had been remarkably free from every annoying symptom; she was educated, and refined in manners, and occupied a position in society, that would induce any physician to be vigilant that no accident should occur, to compromise her recovery.

This physician was called in consultation to deliver a placenta which had been retained, a long time after the birth of the child. Without much difficulty the decomposing and fetid placenta was removed; the hands and arm of the operator washed in soap and water with care; still the noxious odor adhered to the surface most tenaciously. In the night following, this physician was called to attend the lady referred to. The labor was perfectly natural, and by no means severe, but in twenty hours from the time of delivery, she was attacked by a severe chill, followed by high febrile movement in the system—tenderness of the abdomen—tympanites, and arrest of the lochia; and although treatment was commenced promptly to arrest the progress of the case, the effort was unavailing, for the patient died on the second day after the chill.

This patient was perfectly healthy; no accident occurred during or subsequent to labor, to account for the attack, yet she died of puerperal fever. Did not that physician have reason to feel that he had communicated the poison to his patient which caused her death? If this were a solitary instance, he might not censure himself, but when we remember that many cases are on record, similar, in every essential particular, the case is quite different.

How is the contagion communicated? through the atmosphere from patients affected with puerperal fever, erysipelas-gangrene, &c.; by the clothing; from the surface of the body; by the breath of the attendant. That the disease is communicable in the manner indicated, your Committee entertain not the slightest doubt; and they feel called upon to state emphatically their convictions, more especially as medical works written in a most fascinating style, inculcate an opposite view.

This leads to the conclusion that preventive means are never to be lost sight of by any one engaged in obstetric practice. The danger of communicating the disease should at once suggest the impropriety of placing parturient females contiguous to any of the diseases which we have indicated, as liable to communicate puerperal fever. A recognition of this principle would render the propriety of constructing large lying-in hospitals, or appropriating extensive wards to the reception of lying-in women, more than problematical. During the time of the writer's visit to Edinburgh, last fall, which was one of great interest, puerperal fever was prevailing there, and whenever it makes its appearance, as it frequently does, in the Royal Infimary, it is of sufficient gravity to give rise to the greatest anxiety in the minds of the accomplished and skilful physicians having charge of the institution, and we could but feel that a lying-in woman, in a cabin, with barely the necessaries for the emergency, might well be envied by the occupant of a large ward, when the disease is prevailing.

Then what is the duty of the physician, in private practice, during an epidemic of the disease? If engaged at all in obstetric practice, manifestly to relinquish all cases of a zymotic nature, and should the disease appear in his patient, to decline further calls of that nature. A physician is not at liberty to expose the unsuspecting to the danger of this disease, and it is difficult to understand, how those who disregard these principles, can satisfy their consciences while violating these conditions.

It is not our intention to prolong this report, by a criticism on the various modes of treatment, which have from time to time been recommended, further than to raise the question of the propriety of abstracting large quantities of blood in the treatment of puerperal fever. Can a disease be removed from the system by venesection, when the whole volume of blood is affected, and has the power of reproducing itself? If we believe this disease belongs to the class of zymotics, the answer is unhesitatingly, no. Nor shall we detail minutely the plan of treatment which has, under our observation, been most successful. We can at most give only an outline sketch, nor would more than this be desirable at the present time. The plan may be indicated under three heads, viz.:—

1st. Neutralize the *matires morbi* in the system, in the uterus, and in the vagina.

2d. To eliminate the disintegrating and effete materials from the system.

3d. To support the vital forces of the system.

The most efficient agents for fulfilling the first indication yet brought into requisition, we believe to be *chlorine* and *bromine*. The former we have already used sufficiently to enable us to speak with confidence of its good effects. In regard to the latter, from a limited experience with it, we are led to hope for the most satisfactory results. These remedies are valuable in every stage of the disease, and especially indicated when the discharges are profuse or offensive. Locally, they may be applied in solution or used in the form of vapor. Applied in either form they are efficient in proportion to the completeness with which they come in contact with the source and seat of the disease. They are to be introduced not only into the *vagina*, but also into the cavity of the *uterus*. We are far from advising a reckless system of intra uterine injections, but introduced with proper precautions, experience in a few cases, has proved them to be not only harmless but highly efficient agents. The mode proposed is, first to wash away all offensive discharges, at least as completely as practicable, then convey the remedy, properly diluted, to the part, gently through a suitable tube. If vapor be used it may be conveyed to the part by the same apparatus.

The use of chlorine in this disease is not new. It has been demonstrated to be, in this and other diseases, not only a deodorizer but a valuable disinfectant.

The local use of bromine in the treatment of puerperal fever was suggested from its acknowledged value as a remedy in erysipelas hospital gangrene, &c.

The *second indication* can best be fulfilled by the use of remedies, believed to possess the power of preventing or arresting the septic influence of the poison, already circulating with the blood, such as the mineral acids—chlorine salts—the bromides—the sulphites of lime and soda, &c.

To effect the third object, anodynes hold an important place, not only to relieve pain and irritation, but in full doses to arrest the rapid tissue metamorphoses that would take place without their use; with these tonics are indispensible, but several of the most powerful tonics have been already indicated to fulfil the second indication; nutricious diet is indispensible, and in many cases stimulants cannot be dispensed with.

Thus far it has been our object,

1st. To bring into immediate contrast, the diverse views which have been entertained of the nature and treatment of this disease.

2d. To express our belief that it is essentially zymotic in its nature.

3d. That it is communicable by the attendant as well as by other means, and

4th. To direct attention especially to the value of *Chlorine* and *Bromine*, not only in limiting the spread of the disease but also as curative agents.

